# Successful re‐implantation of eroded penile prostheses: Report of two cases and review of the literature

**DOI:** 10.1002/ccr3.8365

**Published:** 2023-12-22

**Authors:** Abbas Basiri, Mazyar Zahir

**Affiliations:** ^1^ Urology and Nephrology Research Center Shahid Beheshti University of Medical Sciences Tehran Iran; ^2^ Erfan Hospital Tehran Iran

**Keywords:** case reports, erosion, penile prosthesis

## Abstract

Prompt removal of eroded penile prostheses is recommended in most cases. However, saving and reimplanting eroded implants may be considered in patients without signs of sepsis, local infection or necrosis during preoperative evaluations and surgical exploration. Notably, close postoperative surveillance is crucial in this setting.

## Graphical abstract

Eroded malleable penile prosthesis.

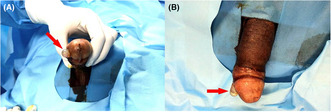

## 1 INTRODUCTION

Since its launch in 1973, penile prosthesis (PP) has significantly improved the management of erectile dysfunction (ED).^1^ While mostly used to treat ED patients who are nonresponsive to phosphodiesterase 5 inhibitors (PDE5i) and intracavernosal injections, PP has also been utilized for Peyronie's disease with simultaneous ED, post‐priapism spongiofibrosis, penile fibrosis, and psychological impotence.^2^

PPs are considered as one of the most successful medical devices due to their outstanding median survival time of approximately 2 decades.^3^ Despite this acceptable performance, device infection and erosion remain as two of the most prevalent complications.^2^, ^4^ PP erosion, defined as an externalization of the prosthetic components, seems to happen due to persistent friction between the prosthetic rods and tunica albuginea over time; ultimately leading to perforation and PP extrusion. In case of extension of the perforation to the overlying skin, erosion ensues.^2^, ^4^ The most common risk factors are attenuated penile sensitivity (e.g., diabetics and paraplegics), vascular diseases, and repeated urethral catheterization.^5^, ^6^ Malleable PPs (MPPs), also known as semi‐rigid PPs, are especially prone to erosion because of their constant erect state.^7^ Nevertheless, erosion can also happen in inflatable PPs (IPPs) and reports have been published on cylinders or connecting tubes eroding through the skin and reservoirs eroding through the bladder, urethra, or even anus.^8^, ^9^, ^10^, ^11^

Current guidelines recommend an instant removal of any eroded component of PPs followed by an immediate or late replacement with a new implant.^4^, ^12^ This approach has been particularly advocated for IPPs to prevent the extension of the possible infection to other compartments of the implant and thus help preserve the remaining parts of the IPP.^12^ While prosthetic removal is inevitable in case of infection, a few studies have suggested that conservative management may be considered in eroded IPPs.^10^ Herein, we report two cases of PP erosion, one in an IPP and the other in a MPP; both re‐implanted with acceptable results.

## 2 CASES

### 2.1 Case 1

**FIGURE 1:**
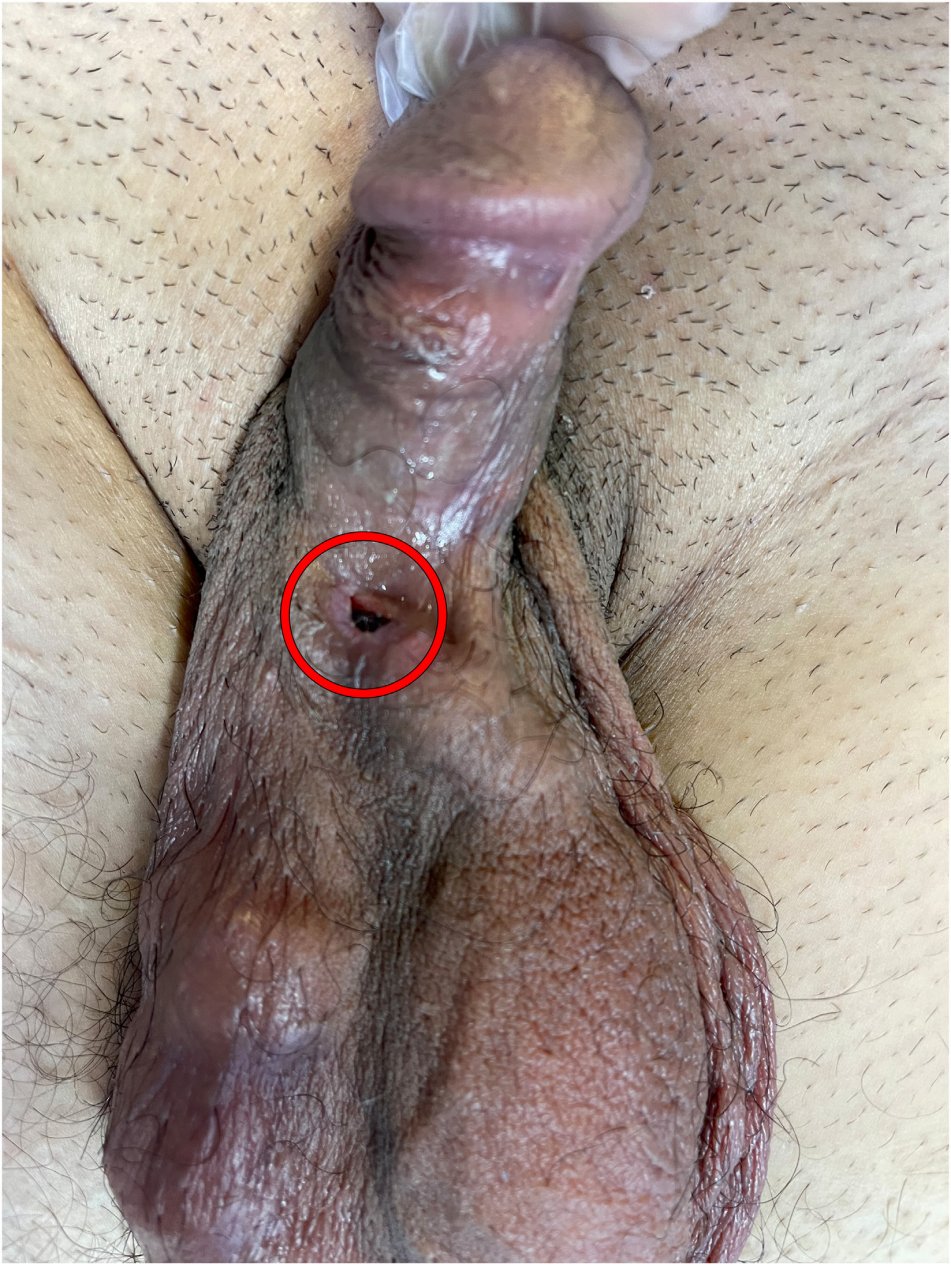
Erosion of the inflatable penile prosthesis tubing system (red circle) into the right penoscrotal region.

**FIGURE 2:**
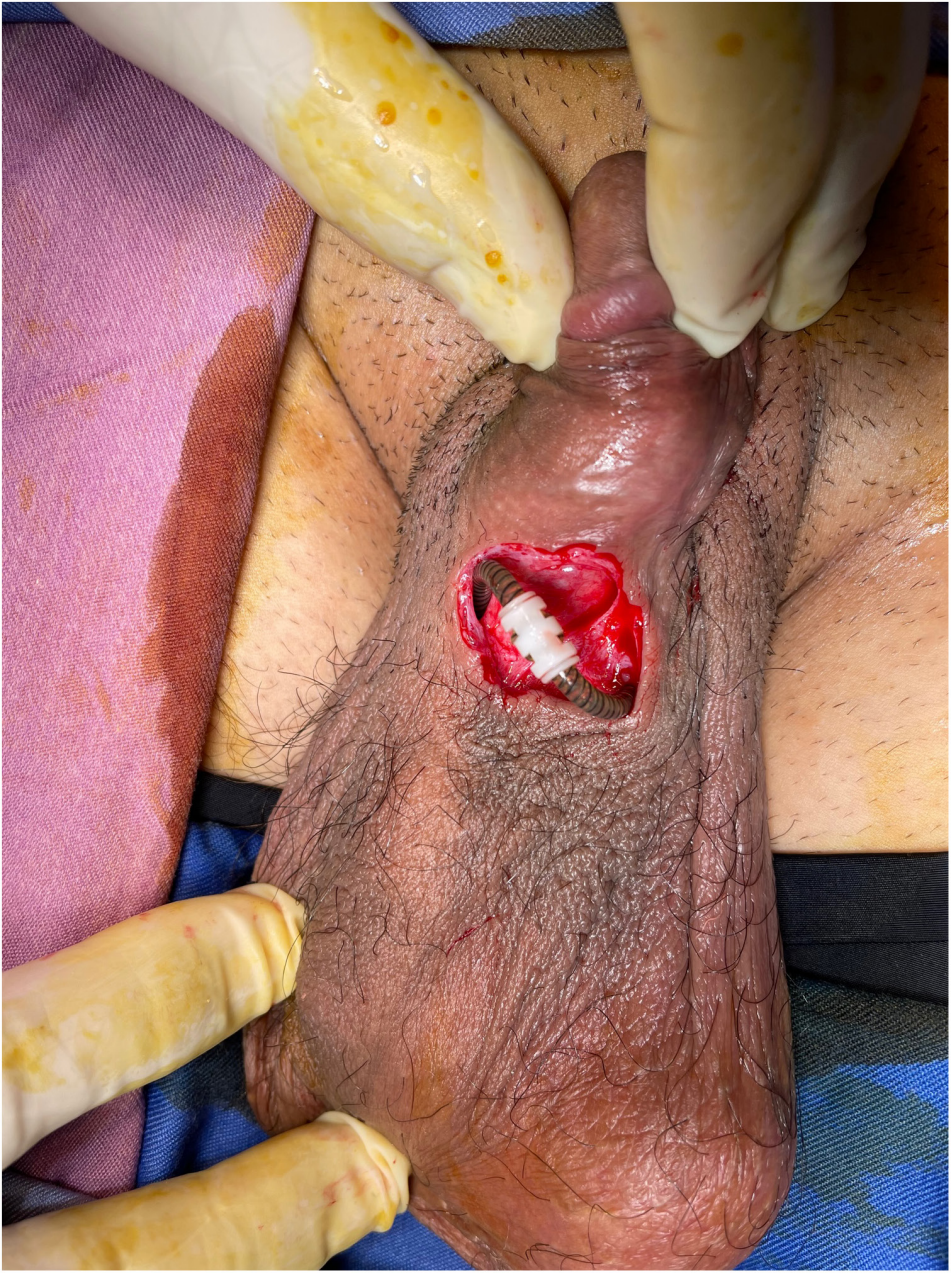
Surgical exploration of the tubing erosion site.

**FIGURE 3:**
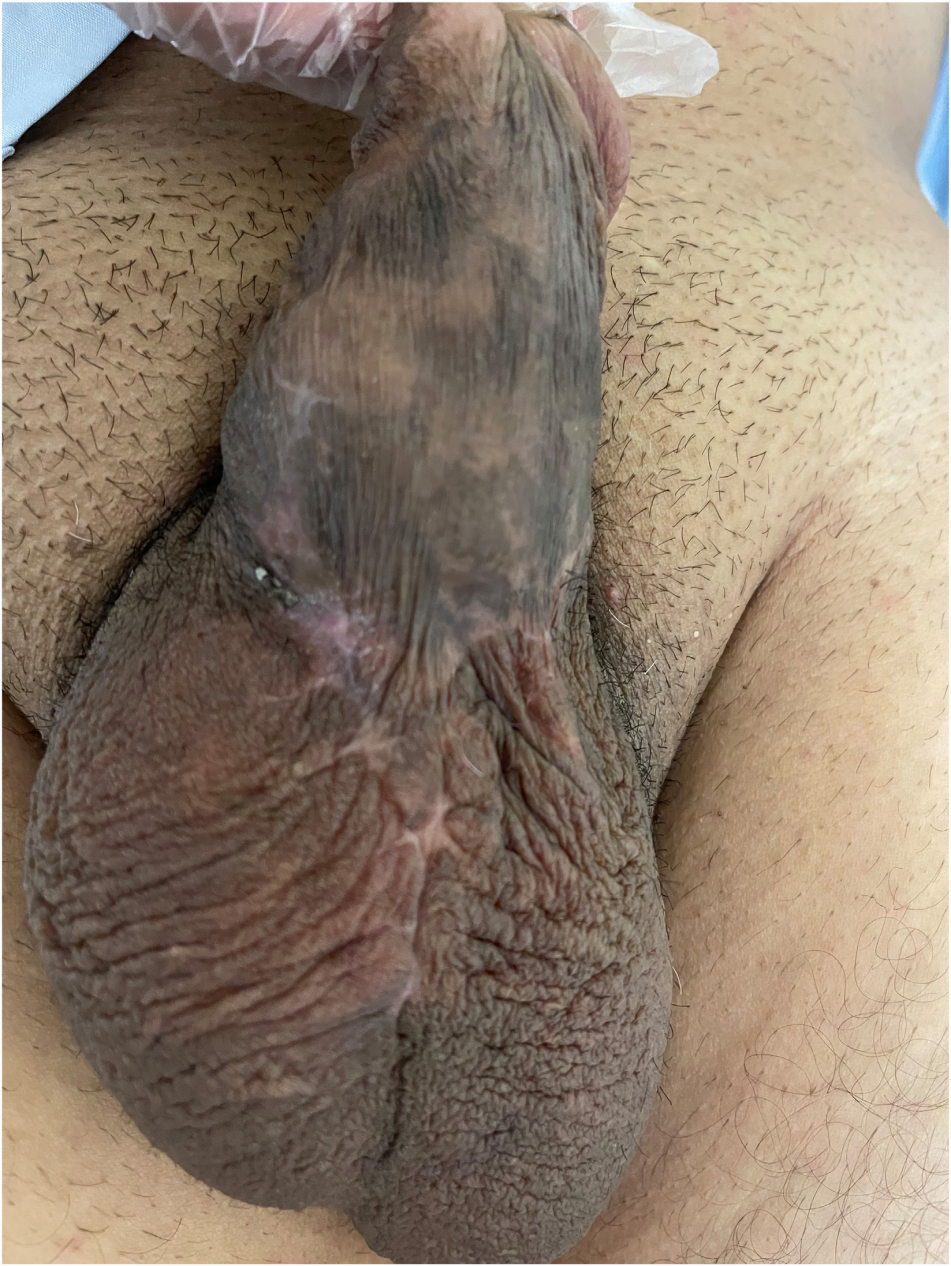
Healing of the erosion site, 2 weeks after surgical exploration and repair.

A 36‐year‐old man with a history of pelvic trauma and no other underlying comorbidity, visited our clinic; complaining of a 2‐year history of ED which happened after his trauma. He was unresponsive to PDE5is, intracavernosal papaverine injections and vacuum constriction device so he was eligible for a 3‐piece IPP (AMS 700™, American Medical Systems Inc., Minnetonka, MN, USA) insertion; which took place with an infrapubic approach without any complications in April 2020. The patient was advised to keep the wound dressing for 3 days and then gently wash the sutured wound with baby shampoo. He was also strongly advised to refrain from sexual intercourse for 2 months. The device had a satisfactory function until February 2021, when the patient noticed the appearance of plastic material in the right penoscrotal region (Figure 1). The patient did not complain from discharge, pain or device malfunction. On examination, the IPP had proper functioning and no sign of pus discharge or inflammation was observed. Microbial culture from tubing and erosion site showed growth of normal skin flora. At surgical exploration, the skin adjacent to the eroded site was excised (Figure 2). The erosion site was then thoroughly explored and irrigated with gentamicin antimicrobial solution and the tube was returned to a deeper pocket. Finally, the overlying skin was sutured. The patient was initially treated with intravenous antibiotics (Imipenem 500 mg, TDS and metronidazole 500 mg, TDS) for 3 days. After his discharge, oral antibiotics (Cephalexin 500 mg, QID and Ciprofloxacin 500 mg, BD) were prescribed for 7 days. Considering the possibility of a false‐negative microbial culture, intraoperative bacterial contamination, an increased risk of infection in redo PP surgery especially when the PP is not removed and notably, the mildly impaired perfusion and sensation of the genital region due to previous trauma, we administered this antibiotic regimen to minimize infection risk. This decision was made after consultation with an infectious diseases specialist and despite our preoperative evaluation showing no evidence of local or systemic infection, and strict adherence to intraoperative infection control measurements. The previously mentioned postoperative precautions were reinstituted for 10 weeks. In the postoperative follow‐up appointment, the surgical incision had healed and the IPP was functioning perfectly (Figure 3). In the last appointment which took place December 2022, the patient had no complaint and the device was fully functional.

### 2.2 Case 2

**FIGURE 4:**
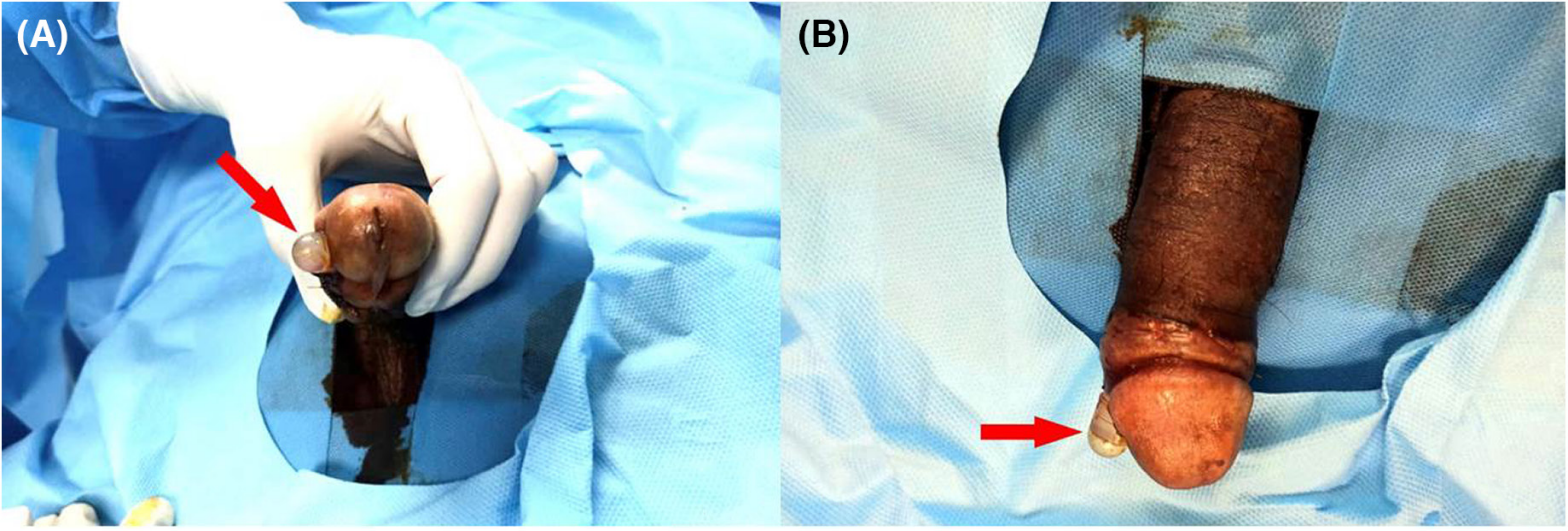
Erosion of the right cylinder tip (red arrow) into the right ventrolateral subcoronal penile surface. (A) frontal view, (B) dorsal view.

**FIGURE 5:**
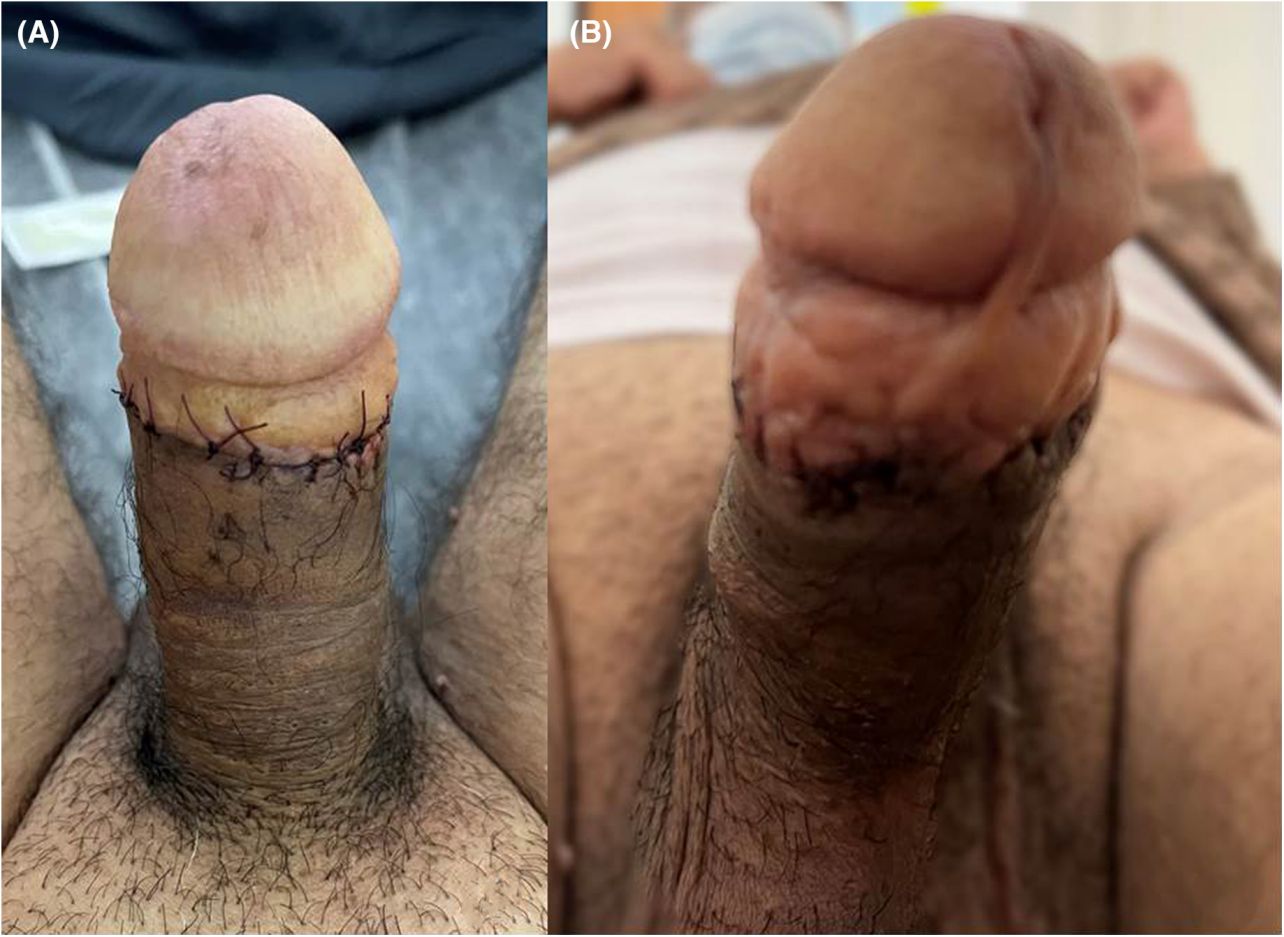
Healing of the erosion site, 1 week after surgical exploration and repair. (A) dorsal view, (B) ventral view.

A 59‐year‐old man with a history of diabetes mellitus and hypertension for a decade was examined in June 2020, complaining of a 3‐year history of vasculogenic ED—diagnosed by Doppler sonography—which did not resolve with PDE5is and intracavernosal papaverine injections. Subsequently, the patient requested a PP and due to financial constraints chose a MPP. An MPP (ZSI 100, Zephyr Surgical implants, Geneva, Switzerland) was utilized for him through an infrapubic incision without any complications in June 2020 and the patient was instructed to maintain the wound dressing for 3 days, followed by a gentle washing of the sutured wound using baby shampoo. He was also strongly advised to abstain from sexual intercourse for 2 months. Nevertheless, in less than 2 months (April 2020), he returned to the emergency department with an erosion of the plastic tip of the right rod into the right ventrolateral subcoronal surface of his penis (Figure 4A,B). The patient complained from mild pain but did not mention any discharge. No sign of purulent discharge or inflammation was noticed on examination. Microbial culture was obtained from erosion site which revealed normal skin flora growth. At surgical exploration, a subcoronal circumferential incision was made and penile skin was degloved and the corpora were incised longitudinally. During exploration it was revealed that the main reason for erosion was a perforation of the distal intercorporal septum located 2 cm proximal to the distal end of the corpora; leading to a corporal crossover of the left rod into the right corporal space and forcing the right rod out of the penile skin at the right ventrolateral subcoronal surface. At first, the rods were removed through the longitudinal corporotomy incisions and the corporal spaces and erosion site were thoroughly irrigated with gentamicin antimicrobial solution. The distal rear tip extenders (RTEs) were then removed and the rods were shortened by 1 cm each. The non‐eroded cylinder was also shortened to ensure equal length of the rods and prevent from uneven rigidity or possible curvature, which can deleteriously affect the appearance and function of the penis. The left corporal space distal to the perforation was then re‐dilated utilizing the *Mulcahy* distal corporoplasty method.^13^ The perforation in the intercorporal septum was double sutured and repaired and the rods were reinserted. The longitudinal corporotomy incisions were also sutured and lastly, the overlying skin was repaired. Initially, the patient was treated with intravenous antibiotics (Imipenem 500 mg, TDS and metronidazole 500 mg, TDS) during his 3 day stay at the hospital. He was discharged thereafter and a 7 day oral antibiotic regimen (Cephalexin 500 mg, QID and Ciprofloxacin 500 mg, BD) was prescribed for him. Considering precautions similar to those of our previous patient, this extensive antibiotic therapy was prescribed after consultation with an infectious diseases specialist who recommended this regimen considering the patient's history of longstanding diabetes and vascular disease, despite both diseases being under tight control. The aforementioned postoperative precautions were reintroduced for 10 weeks. In the follow up visit, surgical incision site was inspected which had healed perfectly (Figure 5A,B). In the last appointment which took place on January 2023 the patient had no complaint and he was satisfied with his PP and the quality of his sexual intercourse.

## 3 DISCUSSION

Previously, a number of case reports have been published on IPP erosions and the subsequent management of this complication. For instance, Kobayashi et al. reported a case of IPP cylinder eroding into the urethral fossa navicularis due to mechanical failure (i.e., autoinflation). They immediately removed the IPP and later replaced it with a MPP.^14^ Likewise, Morgan et al. described a patient who was diagnosed with urethral erosion of the tubing system after several unsuccessful attempts for catheterization; ultimately leading to a removal of the IPP.^9^ Similarly, Panuganti et al. reported three COVID‐19 patients with urethral erosion of IPPs due to prolonged catheterization during their ICU stay leading to removal of the PPs.^15^ In case of intravesical erosion of reservoir, the recommendations are similar; encouraging immediate removal of the reservoir, repairing the vesical walls and replacement with a new reservoir in an alternative location.^8^, ^16^, ^17^ Nevertheless, Izol et al. utilized an alternative approach and showed that the eroded reservoir can be effectively reused and relocated after being profusely irrigated with a disinfectant antimicrobial solution.^18^

Patients with scrotal erosion of the pump or connecting tube often have a milder clinical course and although most clinicians favor removal of the entire device and replacing it with a MPP,^19^ Morales et al. argued that a conservative approach is permissible in the absence of infection and necrosis.^10^ Likewise, Talib et al. suggested that small scrotal erosions can be managed conservatively at first and surgery can be reserved for patients with recurrent erosions unresponsive to conservative management.^20^ Our IPP patient (Case 1) had a relatively similar situation with normal laboratory evaluations and no sign of sepsis, local infection or necrosis in physical examination and surgical exploration; thus allowing conservative management.

As mentioned above, erosions ought to be more common with MPPs. Back in late 1990s, Swana et al. described a quadriplegic patient with an erosion of his MPP into the bladder, necessitating removal of the device.^21^ Owing to the dramatic improvement of MPPs since then, nowadays such complications are much less commonly observed and are almost exclusive to patients with multiple underlying diseases. For instance, in a recent report of a MPP eroding into the urethra; the patient had long‐standing quadriplegia, diabetes, recurrent penile cellulitis, and history of a traumatic catheterization.^22^ Our patient's (Case 2) underlying comorbidities were tightly controlled which led to the assumption that his complication must have been due to intercorporal septal perforations during the initial PP implantation. Although advised to abstain from intercourse for 2 months, he did not provide any information regarding his sexual activity, leading to the possibility that the perforations may have been enlarged during intercourse, resulting in complete corporal crossover and subsequent erosion. After consultating with an internist and an infectionist, who approved his relatively healthy status, and considering the absence of infection or necrosis in surgical exploration, we decided to save his PP during surgery, treat him with antibiotics and keep him under close observation. Fortunately, he fully recovered without any complications.

## 4 CONCLUSIONS

In developing countries like ours, the relative expensiveness and inaccessibility of PPs hinder the immediate decision of their removal in case of erosion without any sign of local or systemic infection. According to our experience, it seems that saving the eroded PP and reimplanting it may be considered in relatively healthy individuals who don't have any sign of sepsis, regional infection and tissue necrosis in physical examination, lab evaluation, and surgical exploration; provided that they remain under close observation after the operation. Finally and most importantly, all patients must be warned to immediately seek medical assistance in case of any sign of extrusion (e.g., observing cylinder tip under penile skin) or obscure pain in genital region to avoid the risk of imminent erosion.

## AUTHOR CONTRIBUTIONS

**Abbas Basiri:** Conceptualization; resources; supervision; writing – review and editing. **Mazyar Zahir:** Data curation; writing – original draft.

## FUNDING INFORMATION

None.

## CONFLICT OF INTEREST STATEMENT

The authors declare that they have no competing interests.

## ETHICS STATEMENT AND CONSENT TO PARTICIPATE

Ethical approval is not required for case reports in our country according to local guidelines. Written consent forms were obtained from the patients.

## CONSENT

Written informed consents were obtained from the patients to publish their health records and relevant accompanying images.

## Data Availability

Not applicable.
